# Multiple internal controls enhance reliability for PCR and real time PCR detection of *Rathayibacter toxicus*

**DOI:** 10.1038/s41598-021-87815-6

**Published:** 2021-04-16

**Authors:** Mohammad Arif, Grethel Y. Busot, Rachel Mann, Brendan Rodoni, James P. Stack

**Affiliations:** 1 Department of Plant and Environmental Protection Sciences, University of Hawaii At Manoa, Honolulu, HI USA; 2 Department of Plant Pathology, Great Plains Diagnostic Network, Kansas State University, 4024 Throckmorton Plant Sciences Center, Manhattan, KS 66506-5502 USA; 3 Department of Economic Development, Jobs, Transport and Resources, Biosciences Research Division, Bundoora, VIC Australia; 4 Plant Biosecurity Cooperative Research Centre, Canberra, Australia; 5 Inari Agricultural Inc., One Kendall Square, Cambridge, MA USA

**Keywords:** Microbiology, Plant sciences

## Abstract

*Rathayibacter toxicus* is a toxigenic bacterial plant pathogen indigenous to Australia and South Africa. A threat to livestock industries globally, the bacterium was designated a U.S. Select Agent. Biosecurity and phytosanitary concerns arise due to the international trade of seed and hay that harbor the bacterium. Accurate diagnostic protocols to support phytosanitary decisions, delineate areas of freedom, and to support research are required to address those concerns. Whole genomes of three genetic populations of *R. toxicus* were sequenced (Illumina MiSeq platforms), assembled and genomic regions unique to each population identified. Highly sensitive and specific TaqMan qPCR and multiplex endpoint PCR assays were developed for the detection and identification of *R. toxicus* to the population level of discrimination. Specificity was confirmed with appropriate inclusivity and exclusivity panels; no cross reactivity was observed. The endpoint multiplex PCR and TaqMan qPCR assays detected 10 fg and 1 fg of genomic DNA, respectively. To enhance reliability and increase confidence in results, three types of internal controls with no or one extra primer were developed and incorporated into each assay to detect both plant and artificial internal controls. Assays were validated by blind ring tests with multiple operators in three international laboratories.

## Introduction

*Rathayibacter toxicus* is a gram-positive bacterial plant pathogen and a Select Agent in the United States since 2008 due to the potential threat to the U.S. livestock industry. Several grass species are hosts for *R. toxicus* including, *Lolium rigidum* Gaudin (annual ryegrass), *Polypogon monspeliensis* (L.) Desf. (annual beard grass), and *Agrostis avenacea* J.F. Gmel. (bent grass or blown-grass)^1,2^. Annual ryegrass toxicity (ARGT) is a disease of livestock and wildlife caused by the ingestion of a toxin excreted by *R. toxicus*. Recurrent livestock deaths due to ARGT outbreaks in Australia have raised biosecurity concerns about the global spread of *R. toxicus.* Inspection and certification of ryegrass-based hay is required prior to export from Australia^3^.

Early detection and accurate diagnosis are essential elements of an effective plant biosecurity system^4,5^. To prevent the introduction of potentially damaging plant pathogenic bacteria into new environments and to mitigate the negative impacts following introduction requires an understanding of pathogen population biology. Currently, three genetic populations of *R. toxicus* have been described: RT-I and RT-II (South Australia) and RT-III (Western Australia)^1^. The newly detected population, RT-I, was the predominant population on the Yorke Peninsula, South Australia in 2014–2016; its prevalence and genetic analyses suggest a more recent emergence^1^. However, no qPCR-based assay has been developed for this high consequence pathogen.

Validated diagnostic assays for the reliable detection of *R. toxics* are needed^6^. TaqMan probe-based qPCR methods are used to detect target pathogen(s) with high sensitivity and reliability for applications in routine diagnostics, microbial forensics and plant biosecurity^7–9^. Multiplex endpoint PCRs are commonly used to differentiate closely related bacterial strains from the same host based on genomic variation^10^ (Strayer et al., 2016). Multiplex endpoint PCR methods are cost effective and easy to perform compared to TaqMan probe based multiplex qPCR methods^11^.

Accuracy and reliability of qPCR and PCR methods are greatly enhanced by using appropriate positive and internal controls^12,13^. Positive controls comprised of a single PCR tube containing the target DNA are commonly used in diagnostic laboratories. However, PCR reactions fail for a variety of reasons, including, deviation from the validated SOP, the presence of PCR inhibitors, equipment performance, or operator error^14,15^. Many PCR failures can’t be detected nor the reason for failure identified using only a single positive control. Therefore, inclusion of a single PCR tube as a positive control cannot determine the reliability and accuracy of PCR results in every reaction tube. For diagnostics requiring high confidence in test results, the inclusion of an internal control(s) in each PCR tube is extremely important to avoid the possibilities of false negative results.

In this research, we developed accurate, reliable and sensitive diagnostic TaqMan probe-based multiplex real time qPCR and multiplex endpoint PCR for reliable detection and discrimination of the Select Agent, *R. toxicus*. We also designed, developed and compared three kinds of internal controls: two customized artificial internal controls and one to target the host genome. The developed tools will provide capabilities for application in routine detection and discrimination of *R. toxicus*, ARGT disease outbreak management, agriculture biosecurity and *R. toxicus* population monitoring. Development of a customized artificial internal control may provide insight to develop AICs for other target pathogens.

## Results

### Primer and probe specificity

**Figure 1:**
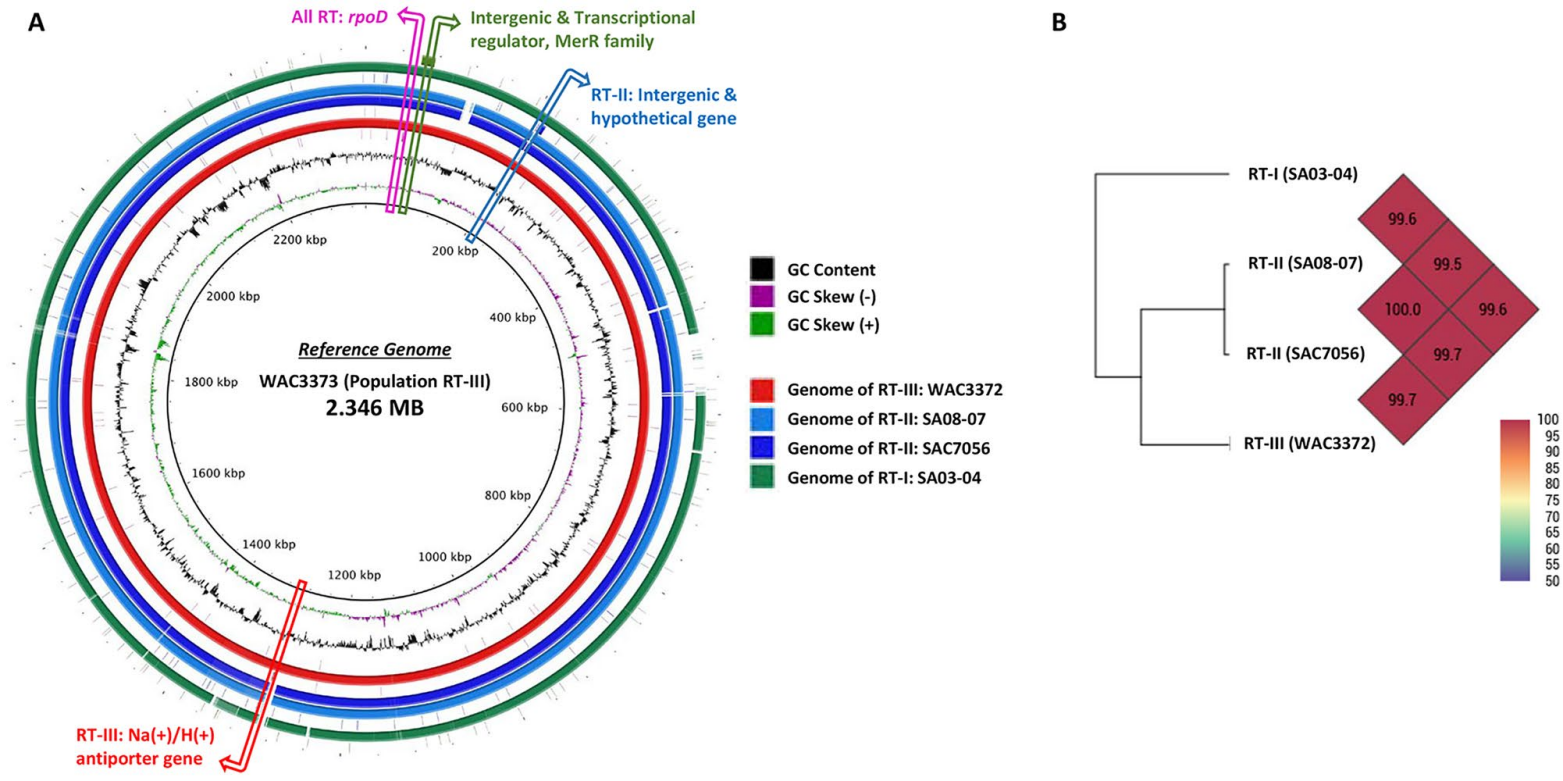
Target genome region selection to design species and population specific primers and probes for *Rathayibacter toxicus*. (**a**) Genomes of isolates SA03-04 (2013; population RT-I; South Australia), SA08-07 (2013; population RT-II; South Australia), SAC7056 (1983; population RT-II; South Australia) and WAC3372 (1978; population RT-III; Western Australia) were aligned against the reference genome of WAC3373 (GenBank accession# NZ_CP013292) and target genomic regions were selected. (**b**) All four genomes were calculated for Average Nucleotide Identity (ANI) and showed maximum 0.5% dissimilarity between genome SA08-07 and SA03-04.

The primer sets designed for *R. toxicus* showed 100% query coverage and 100% identity with their corresponding sequence targets. The genomic regions unique to three populations (RT-I, RT-II and RT-III) of *R*. *toxicus* were identified by aligning the genomes from all three populations (Fig. 1). Both NCBI GenBank nucleotide database *R. toxicus* genome database were used to confirm, in silico*,* the identity of each primer and probe used in this study; all showed high specificity with their corresponding target. Delta G for each primer and probe calculated for internal structures at 60 °C using mFold showed delta G value less than 1.0, an indicator of good primer and probe thermodynamics.

### Primers and probe specificity with members of the inclusivity and exclusivity panels

Primers and probes were designed for TaqMan real-time qPCR and endpoint PCR to detect and discriminate among populations of *R. toxicus*. Multiplex endpoint PCR specifically discriminated all three reported populations of *R. toxicus* viz. RT-I, RT-II and RT-III. Each primer set used in qPCR and endpoint PCR was tested for specificity against an inclusivity panel which comprised 54 isolates of *R. toxicus* collected during the last 4 decades and from different geographical regions of Australia (Table 1). The primer and probe sets for qPCR and endpoint PCR developed for general detection of all *R. toxicus* populations showed 100% amplification with all 54 isolates (Tables 1, 2). However, primer sets that were designed to detect only a specific population of *R. toxicus,* detected only that target population when used in endpoint multiplex PCR. To determine the potential for false positives, all primers and probe were tested against an exclusivity panel which included the taxonomically close relatives, *R. tritici, R. iranicus, R. rathayi,* and *R. agropyri* as well as the ecologically-associated *D. cinnemea*; no cross reactivity was observed with any member of the exclusivity panel. No cross reactivity was observed with healthy plant host (*L. rigidium*, annual ryegrass) DNA. The assays were tested for specific detection of *R. toxicus* from infected annual ryegrass samples collected in 2014 from South Australia and Western Australia; both qPCR and endpoint PCR assays specifically detected *R. toxicus* from the known infected samples (Supplemental Table 1). Samples obtained from location SA19 (South Australia)^1^ showed the presence of population RT-I and RT-II when a multiplex endpoint PCR was performed to detect and discriminate *R. toxicus* populations. Specificity of all assays remained consistent across all four master mix kits tested.

**Table 1:** Composition of inclusivity and exclusivity panels to confirm specificity of multiplex endpoint PCR and TaqMan qPCR to detect and discriminate *Rathayibacter toxicus.*

Genus/species	Isolate code	Population type	Host	**Yr	Location/source	Detection results		
						qPCR	Multiplex PCR	Population identified using multiplex PCR
*Rathayibacter toxicus*	SA03-02	RT-I	ARG	2014	Corny point, SA	+	+	RT-I
*R. toxicus*	SA03-03	RT-I	ARG	2014	Corny point, SA	+	+	RT-I
*R. toxicus*	SA03-04	RT-I	ARG	2014	Corny point, SA	+	+	RT-I
*R. toxicus*	SA03-08	RT-I	ARG	2014	Corny point, SA	+	+	RT-I
*R. toxicus*	SA03-14	RT-I	ARG	2014	Corny point, SA	+	+	RT-I
*R. toxicus*	SA03-15	RT-I	ARG	2014	Corny point, SA	+	+	RT-I
*R. toxicus*	SA03-16	RT-I	ARG	2014	Corny point, SA	+	+	RT-I
*R. toxicus*	SA03-17	RT-I	ARG	2014	Corny point, SA	+	+	RT-I
*R. toxicus*	SA03-18	RT-I	ARG	2014	Corny point, SA	+	+	RT-I
*R. toxicus*	SA03-19	RT-I	ARG	2014	Corny point, SA	+	+	RT-I
*R. toxicus*	SA03-20	RT-I	ARG	2014	Corny point, SA	+	+	RT-I
*R. toxicus*	SA03-21	RT-I	ARG	2014	Corny point, SA	+	+	RT-I
*R. toxicus*	SA03-22	RT-I	ARG	2014	Corny point, SA	+	+	RT-I
*R. toxicus*	SA03-23	RT-I	ARG	2014	Corny point, SA	+	+	RT-I
*R. toxicus*	SA03-24	RT-I	ARG	2014	Corny point, SA	+	+	RT-I
*R. toxicus*	SA03-25	RT-I	ARG	2014	Corny point, SA	+	+	RT-I
*R. toxicus*	SA03-26	RT-I	ARG	2014	Corny point, SA	+	+	RT-I
*R. toxicus*	SA03-27	RT-I	ARG	2014	Corny point, SA	+	+	RT-I
*R. toxicus*	SA03-28	RT-I	ARG	2014	Corny point, SA	+	+	RT-I
*R. toxicus*	SA08-03	RT-I	ARG	2014	Lake sunday, SA	+	+	RT-I
*R. toxicus*	SA08-07	RT-II	ARG	2014	Lake sunday, SA	+	+	RT-II
*R. toxicus*	SA08-08	RT-I	ARG	2014	Lake sunday, SA	+	+	RT-I
*R. toxicus*	SA08-09	RT-I	ARG	2014	Lake sunday, SA	+	+	RT-I
*R. toxicus*	SA08-11	RT-I	ARG	2014	Lake sunday, SA	+	+	RT-I
*R. toxicus*	SA08-13	RT-I	ARG	2014	Lake sunday, SA	+	+	RT-I
*R. toxicus*	SA08-16	RT-I	ARG	2014	Lake sunday, SA	+	+	RT-I
*R. toxicus*	SA19-02	RT-I	ARG	2013	Yorketown, SA	+	+	RT-I
*R. toxicus*	SA19-03	RT-II	ARG	2013	Yorketown, SA	+	+	RT-II
*R. toxicus*	SA19-04	RT-I	ARG	2013	Yorketown, SA	+	+	RT-I
*R. toxicus*	SA19-05	RT-II	ARG	2013	Yorketown, SA	+	+	RT-II
*R. toxicus*	SA19-06	RT-I	ARG	2013	Yorketown, SA	+	+	RT-I
*R. toxicus*	SA19-07	RT-I	ARG	2013	Yorketown, SA	+	+	RT-I
*R. toxicus*	SA19-08	RT-II	ARG	2013	Yorketown, SA	+	+	RT-II
*R. toxicus*	SA19-09	RT-I	ARG	2013	Yorketown, SA	+	+	RT-I
*R. toxicus*	SA19-10	RT-II	ARG	2013	Yorketown, SA	+	+	RT-II
*R. toxicus*	SA19-11	RT-I	ARG	2013	Yorketown, SA	+	+	RT-I
*R. toxicus*	SA19-12	RT-I	ARG	2013	Yorketown, SA	+	+	RT-I
*R. toxicus*	SA19-13	RT-I	ARG	2013	Yorketown, SA	+	+	RT-I
*R. toxicus*	SA19-14	RT-II	ARG	2013	Yorketown, SA	+	+	RT-II
*R. toxicus*	SAC3368	RT-II	ARG	1981	SA	+	+	RT-II
*R. toxicus*	SAC3387	RT-II	ARG	1981	SA	+	+	RT-II
*R. toxicus*	SAC7056	RT-II	ARG	1983	Murray Bridge, SA	+	+	RT-II
*R. toxicus*	WAC3371	RT-III	LCG	1978	Gnowangerup, WA	+	+	RT-III
*R. toxicus*	WAC3372	RT-III	BO	1978	Gnowangerup, WA	+	+	RT-III
*R. toxicus*	WAC3373	RT-III	PG	1978	Gnowangerup, WA	+	+	RT-III
*R. toxicus*	WAC3396	RT-III	Oat	1980	Gnowangerup, WA	+	+	RT-III
R. toxicus*	FH100	RT-II	ABS	1991	Southeastern, SA	+	+	RT-II
R. toxicus*	FH83	RT-II	ARG	1975	SA	+	+	RT-II
R. toxicus*	FH85	RT-II	ARG	1975	SA	+	+	RT-II
R. toxicus*	FH147	RT-II	ARG	1984	SA	+	+	RT-II
R. toxicus*	FH141	RT-II	ARG	1983	SA	+	+	RT-II
R. toxicus*	FH81	RT-III	ARG	1973	WA	+	+	RT-III
R. toxicus*	FH138	RT-III	ARG	1978	WA	+	+	RT-III
R. toxicus*	FH87	RT-III	ARG	1974	WA	+	+	RT-III
*R. tritici*	WAC7055	–	Wheat	1991	Carnamah, WA	–	–	–
*R. tritici*	WAC9601	–	RG	–	South Perth, WA	–	–	–
*R. tritici*	WAC9602	–	RG	–	South Perth, WA	–	–	–
*R. agropyri*	WAC9620	–	RG	–	South Perth, WA	–	–	–
*R. agropyri*	WAC9594	–	RG	–	South Perth, WA	–	–	–
*R. iranicus*	ICMP 12,831	–	Wheat	1994	Iran	–	–	–
*R. iranicus*	ICMP 13,126	–	Wheat	1994	Iran	–	–	–
*R. iranicus*	ICMP 13,127	–	Wheat	1994	Iran	–	–	–
*R. iranicus*	ICMP 3496	–	Wheat	1996	Iran	–	–	–
*R. rathayi*	ICMP 2579	–	DG	–	United Kingdom	–	–	–
*R. rathayi*	ICMP 2574	–	DG	1968	New Zealand	–	–	–
*R. rathayi*	WAC3369	–	ARG	–	WA	–	–	–
*Dietzia cinnamea*	SA03-14 M	–	ARG	2014	Corny Point, SA	–	–	–
Host DNA			ARG					

*Only DNA was available for this study; *AIC* artificial internal control; **Yr—year of collection.

**Table 2:** Characteristics of primers used to differentially detect three *Rathayibacter toxicus* populations, host *Lolium rigidum* (host internal control) and plasmid DNA (internal controls).

Primer name	Primer sequence (5′-3′)	Target	Target gene	Length	%GC	*ΔG	^any	^^3	Amplicon size (bp)
RT-F1 RT-R1 RT-R3	AGAACTTTCGCGAGAACTCGAT AAGCGCATCCGAATCACC GAATTCGCTATCGCCGTCTT	*R. toxicus*	*rpoD*	22 18 20	45 56 50	0.9 0.0 0.6	6 4 6	2	RT-F1/R1 = 246 RT-F1/R3 = 115
								0	
								2	
RT-AF1 RT-AR1	ATAGGAGCCCGGTAATGGAAT CCTCAACCTCATAGCGGTCAT	*R. toxicus,* RT-I	Intergenic & Transcriptional regulator, MerR family	21 21	48 52	0.0 0.0	4 4	2 2	RT-AF1/AR1 = 411
RT-BF1 RT-BR1	TAGGCAGCAGTTTCTTCACCA GGCGTACCTTCAGGAATCATC	*R. toxicus*, RT-II	Intergenic & hypothetical gene	21 21	48 52	0.9 0.0	3 4	0 1	RT-BF1/BR1 = 532
RT-CF1 RT-CR1	GACTCTCGTGGCTGATTGATG TTACTCTCTGGGTGGGTCTGC	*R. toxicus*, RT-III	Na( +)/H( +) antiporter gene	21 21	52 57	0.9 0.8	3 2	0 2	RT-CF1/CR1 = 677
IC-PF2 IC-PR2	ACTCTCGGCAACGGATATCTC GCAACTTGCGTTCAAAGACTC	Host	ITS	21 21	52 48	0.0 0.9	6 7	4 3	72^a^ or 113^b^

*Plot ΔG value in kcal/mol calculated by mFOLD; ^max self-complementarity; ^^max 3′ self-complementarity; *rpoD*—RNA polymerase sigma factor *rpoD;* RT-I, RT-II & RT-III are *R. toxicus* populations; ^a^amplified a 72 bp fragment when used with artificial internal control AIC-2 contains targets for primer IC-PF2, IC-PR2; (c) ^b^amplified a 113 bp fragment when used with host DNA (host internal control – HIC).

### Endpoint PCR sensitivity assays

**Figure 2:**
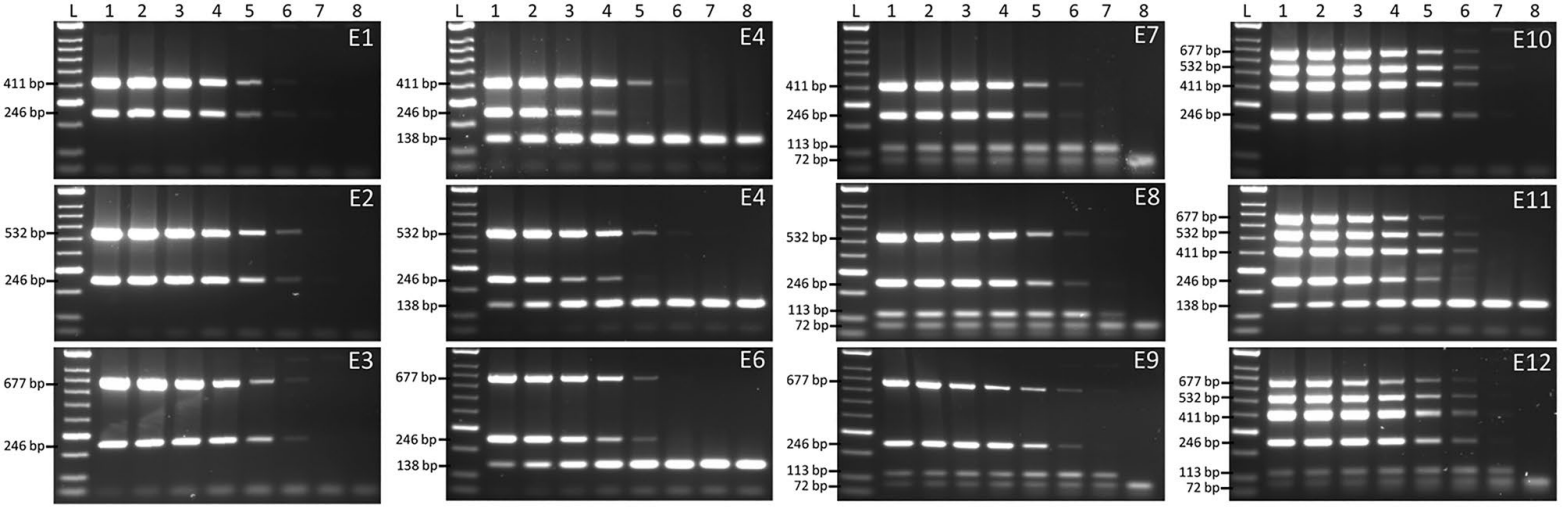
Comparative sensitivity assays to predict the detection limit of *Rathayibacter toxicus* using multiplex endpoint PCR with and without internal controls. Lane 1–7 are tenfold serial dilutions of genomic DNA from 1 ng to 1 fg; lane 8 is non-template control (NTC; water). Qiagen multiplex kit was used for these sensitivity assays. E1. DNA from population RT-I only + No AIC; E2. DNA from population RT-II only + No AIC; E3. DNA from population RT-III only + No AIC; E4. DNA from population RT-I only + AIC-1; E5. DNA from population RT-II only + AIC-1; E6. DNA from population RT-III only + AIC-1; E7. DNA from population RT-I only + AIC-2 & HIC; E8. DNA from population RT-II only + AIC-2 & HIC; E9. DNA from population RT-III only + AIC-2 & HIC; E10. DNA from all three populations + No AIC; E11. DNA from all three populations + AIC-1; E12. DNA from all three populations + AIC-2 & HIC.

Sensitivity assays were performed with and without internal controls for single target and multiplex assays (Supplemental Table 2). Endpoint multiplex PCR could detect down to 10 fg (Fig. 2 & Supplemental Fig. 1, 2). With Qiagen Multiplex kit, no effect on sensitivity was observed when AIC-2 and HIC was added in the reaction. However, addition of AIC-1 with no additional primer reduced the sensitivity up to 100-fold (Fig. 2E4) when only single population DNA was used; a ten-fold affect was observed in multiplex PCR with all *R. toxicus* populations (Fig. 2E11). With GoTaq Green Master Mix kit, the single target, population specific reactions showed sensitivity to 10 fg; primer set RT-F1/RT-R1 was able to detect only to 100 fg. This kit also successfully detected target DNA up to 10 fg but like the Qiagen Multiplex Kit, it didn’t work very well when AIC-1 was added in the reaction with no additional primer set; sensitivity affected 10,000-fold. Overall, the Qiagen Multiplex Kit performed better when AIC-1 was added in the reaction. Both kits showed no difference in sensitivity when multiplex assays with all DNA and with AIC-2 and HIC were performed (Fig. 2, Supplemental Fig. 2). Endpoint multiplex sensitivity results were consistent with Qiagen Multiplex Kit compared to GoTaq Green Master Mix. A total of 28 endpoint PCR sensitivity assays were performed to compare the sensitivity of developed assays (Supplemental Table 2).

### TaqMan real-time qPCR sensitivity assays

**Figure 3:**
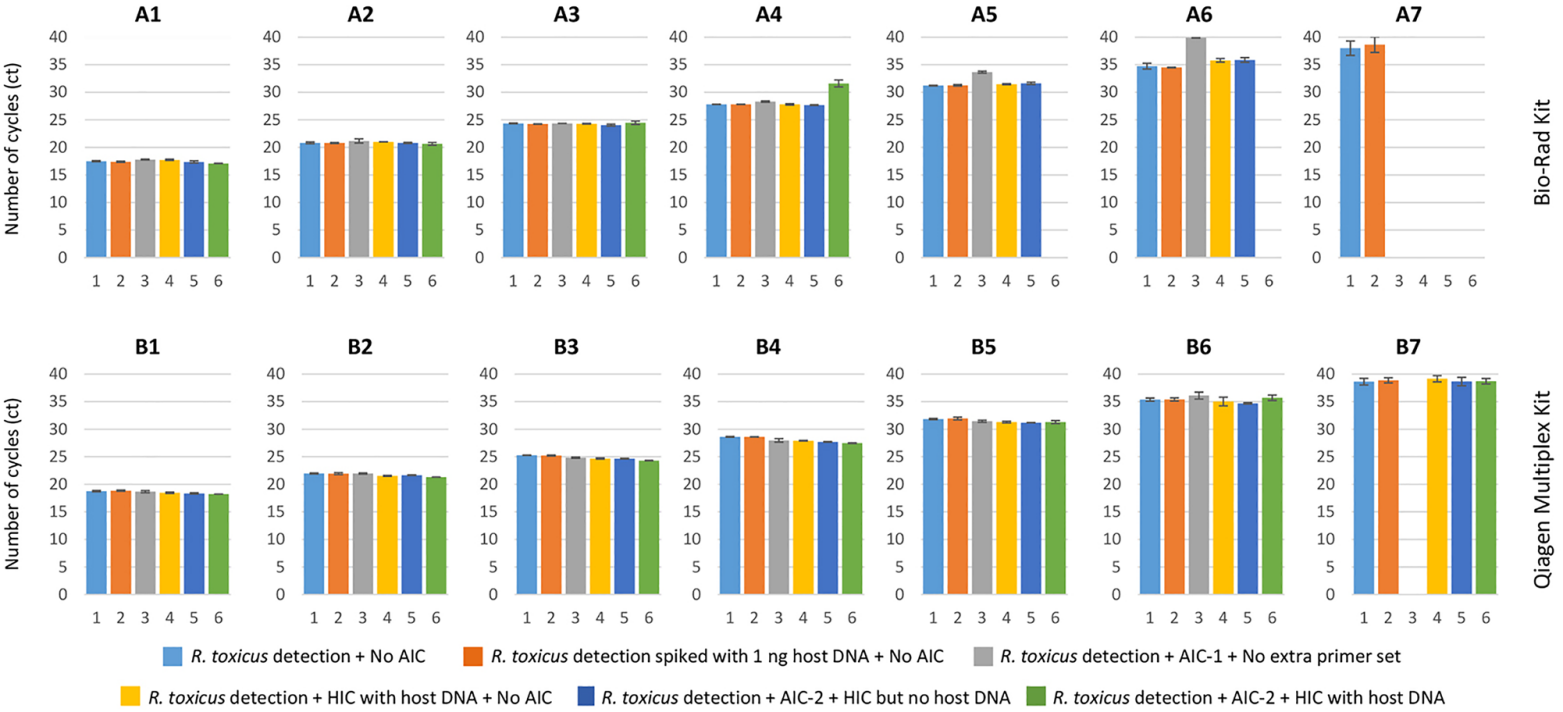
Comparative sensitivity assays to predict the detection limit of TaqMan qPCR-based assays with genomic and spiked DNA of *Rathayibacter toxicus*. A1–A7 are tenfold serial dilutions of genomic DNA from 1 ng to 1 fg and performed using Bio-Rad SsoFast Probes Supermix Kit; B1-B7 are genomic DNA dilutions from 1 ng to 1 fg and performed using Rotor-Gene Multiplex PCR Master Mix Kit. Standard deviation was calculated using three replicates of each reaction and each PCR assay was run with non-template negative control.

**Figure 4:**
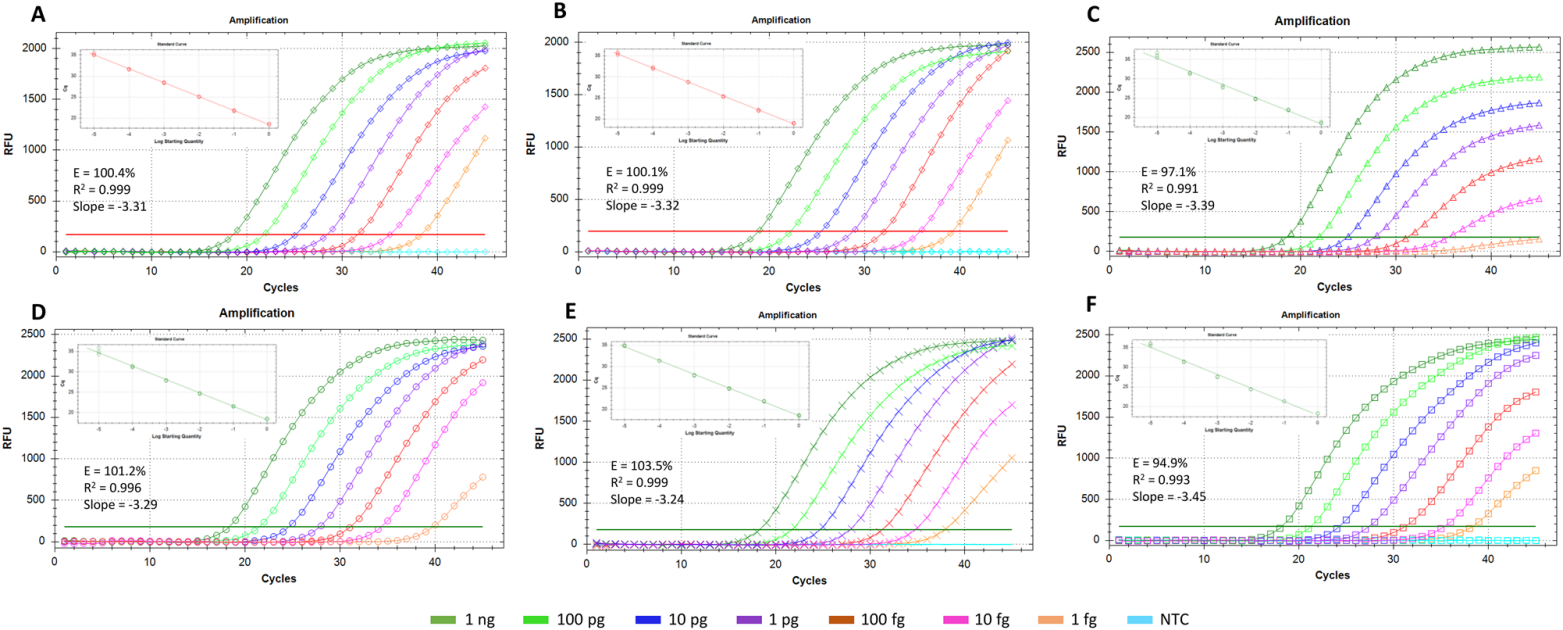
TaqMan real-time qPCR-based sensitivity assays with and without internal controls to predict detection limit of *Rathayibacter toxicus*. Qiagen Rotor-Gene Multiplex PCR Master Mix was used for these sensitivity assays. (**A**) *R. toxicus* detection + No AIC; (**B**) *R. toxicus* detection spiked with 1 ng host DNA + No AIC; (**C**) *R. toxicus* detection + AIC-1 + No additional primer set was added (only *R. toxicus* specific primers were used); (**D**) *R. toxicus* detection + HIC with host DNA + No AIC; (**E**) *R. toxicus* detection + AIC-2 + HIC but no host DNA; (**F**) *R. toxicus* detection + AIC-2 + HIC with host DNA. Dilution color codes are provided at the bottom of the figure; NTC is non-template control (water).

**Figure 5:**
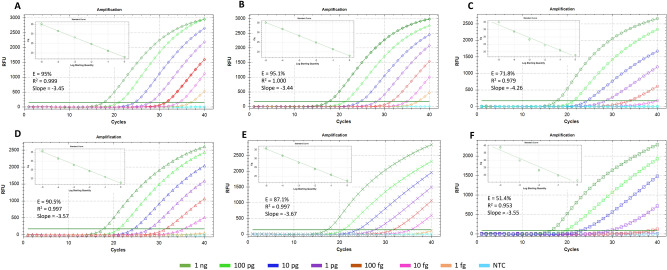
TaqMan real-time qPCR-based sensitivity assays with and without internal controls to predict detection limit of *Rathayibacter toxicus*. Bio-Rad SsoFast Probes Supermix was used for these sensitivity assays. (**A**) *R. toxicus* detection + No AIC; (**B**) *R. toxicus* detection spiked with 1 ng host DNA + No AIC; (**C**) *R. toxicus* detection + AIC-1 + No additional primer set was added (only *R. toxicus* specific primers were used); (**D**) *R. toxicus* detection + HIC with host DNA + No AIC; (**E**) *R. toxicus* detection + AIC-2 + HIC but no host DNA; (**F**) *R. toxicus* detection + AIC-2 + HIC with host DNA. Dilution color codes are provided at the bottom of the figure; NTC is non-template control (water).

The TaqMan qPCR assays were also performed with and without internal controls (AIC-1, AIC-2 and HIC) and detected down to 1 fg of target *R. toxicus* DNA. Multiplex TaqMan qPCR results were consistent and showed optimal reaction efficiency (ca. 100%), R^2^ (ca. 1.00) and slope value (ca. 3.3) with Rotor-Gene Qiagen Multiplex Kit compared to Bio-Rad SsoFast Probes Supermix (Figs. 3, 4, 5). However, Bio-Rad SsoFast Probes Supermix worked well with single target (Figs. 3, 4, 5). Qiagen multiplex showed no effect with and without internal controls except with AIC-1 internal control where sensitivity was affected by10-fold (Fig. 4).

### Internal controls

**Figure 6:**
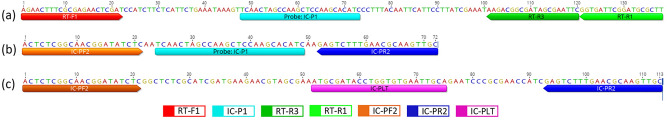
Internal control target regions and design – both AIC-1 and AIC-2 were artificially synthesized. (**a**) Artificial internal control AIC-1 contains target for primer RT-F1, RT-R1, RT-R3 and a unique probe IC-P1; (**b**) artificial internal control AIC-2 contains target for primer IC-PF2, IC-PR2 and a unique probe IC-P1; (**c**) host internal control HIC is an internal transcribed spacer sequence of host genome (*Lolium rigidum*) contains target for primers IC-PF2, IC-PR2 and probe IC-PLT. AIC-1 can be detected by using the same primer as for *R. toxicus*—no need to add another set of primer but require an additional unique probe to be detected by different channel of qPCR. AIC-2 can be detected by using the same primer set as to detect host genome—no need to add any additional primer set to detect AIC-2 but require an additional unique probe to be detected by different qPCR channel. An additional primer and probe is required for HIC.

Three different types of internal controls were used to enhance the reliability and accuracy of the assays. Unique approaches were used to design these internal controls. The AIC-I internal control did not require an additional primer set in the reaction; primer set RT-F1 and RT-R1/RT-R3 detected both internal control and all populations of *R. toxicus*. Although the AIC-2 internal control required an additional primer set to detect AIC-2, the same primer set also detected host DNA (ITS; Internal Transcribed Spacer region). The third internal control detected only host DNA. All internal controls worked well; AIC-2 and HIC had no effect on sensitivity, but AIC-1 decreased sensitivity in the endpoint multiplex PCR and TaqMan qPCR reactions (Figs. 2, 3, 4) when Qiagen Multiplex kits were used. However, the addition of any internal controls negatively affected the sensitivity and reaction efficiency when the Bio-Rad SsoFast Probes Supermix kit was used in the multiplex TaqMan real-time qPCR (Fig. 5). Two different probes were used to detect the different internal controls (Fig. 6; Table 3).

**Table 3:** Characteristics of probes used in detection assays of *Rathayibacter toxicus*, host *Lolium rigidum* (host internal control) and plasmid DNA (artificial internal controls).

Probe name	Probe sequence (5′-3′)	Target	Target Gene	Length	%GC	*ΔG	^any	^^3	Reporter dye	Quencher dye	EX Spectra (nm)	EM Spectra (nm)	qPCR Channel
RT-P1	TCGTGGAAGTACAAAAGTATGGTCGC	*R. toxicus*	*rpoD*	26	46	0.7	4	2	6-FAM	ZEN-IABkFQ	495	520	Green
IC-PLT	ATGCGATACCTGGTGTGAATTGCA	HIC	ITS	24	46	0.8	5	4	6-ROXN	BHQ2	575	602	Orange
IC-P1	TCAACTAGCCAAGCTCCAAGCACATC	AIC-1 & AIC-2	–	26	50	0.0	4	0	Cy5	IAbRQSp	647	667	Red

*Plot ΔG value in kcal/mol calculated by mFOLD; ^max self-complementarity; ^^max 3′ self-complementarity; *rpoD*—RNA polymerase sigma factor *rpoD;* BHQ2-Black Hole Quencher 2; IAbRQSp- Iowa Black RQ-Sp; IABkFQ-Iowa Black FQ; HIC-host internal control; AIC-artificial internal control.

### Blind ring tests and multiple operators

Blind ring tests were performed by multiple operators in five labs of three countries, namely, Australia, New Zealand and United States. The blind ring test panel comprised of eight samples that included DNA extractions from the different *R. toxicus* populations and a tube of sterile water, were sent to each lab with the protocols provided in this manuscript. The results obtained from each lab were specific and concordant with the expected/predicted results; an indication of the robustness and reliability of each developed method (Table 4).

**Table 4:** Blind test results of the *Rathayibacter toxicus* specific assays performed by multiple operators in multiple national and international labs.

Pathogen	Isolate number	Performed by 1^st^ Author at Kansas State University, Manhattan, KS, USA			Performed at Oklahoma State University, Stillwater, OK, USA		Performed at Elizabeth Macarthur Agricultural Institute, Menangle, NSW, Australia		Performed at La Trobe University, Bundoora, VIC, Australia	Plant & Food Research, Lincoln, New Zealand		
		qPCR	mPCR	Identified population	mPCR	Identified population	mPCR	Identified population	qPCR	qPCR	mPCR	Identified population
*Rathayibacter toxicus*	SA03-25	+	+	RT-I	+	RT-I	+	RT-I	+	+	+	RT-I
*R. toxicus*	SA19-03	+	+	RT-II	+	RT-II	+	RT-II	+	+	+	RT-II
*R. toxicus*	SA19-02	+	+	RT-I	+	RT-I	+	RT-I	+	+	+	RT-I
Water	Water	–	–	–	–	–	–	–	–	–	–	–
*R. toxicus*	SA19-09	+	+	RT-I	+	RT-I	+	RT-I	+	+	+	RT-I
*R. toxicus*	WAC3373	+	+	RT-III	+	RT-III	+	RT-III	+	+	+	RT-III
*R. toxicus*	SA08-07	+	+	RT-II	+	RT-II	+	RT-II	+	+	+	RT-II
*R. toxicus*	SAC3387	+	+	RT-II	+	RT-II	+	RT-II	+	+	+	RT-II

These assays were performed with internal control at different time intervals; assays at Oklahoma State University, La Trobe University, Elizabeth Macarthur Agricultural Institute and Plant & Food Research on 12/2014, 11/2015, 12/2015 and 12/2015, respectively. The assays were repeated twice by 1^st^ author as well. mPCR is multiplex PCR; qPCR is quantitative TaqMan PCR.

## Discussion

In this paper, we not only report the development of robust, population-specific and highly sensitive multiplex endpoint PCR and TaqMan qPCR assays for detection of the high consequence Select Agent, *R. toxicus*, but we also describe the development and validation of a novel set of internal controls to enhance confidence in assay results. The internal controls were designed to provide quality assurance for the different stages of a PCR-based diagnostic protocol (Table 5).

**Table 5:** Value of internal controls to increase confidence in PCR and qPCR-based diagnostics.

Potential PCR errors		PCR controls	
Error	Cause	Solution	Test Purpose
False positives	Cross contamination	No-template control (negative control)	To verify no cross contamination
	Primer mismatch	Non-target DNA (near relative)	To verify specificity of primers
	Faulty equipment (e.g., inaccurate run temperatures)	Target DNA (positive control)	To verify primer, probe and reagent efficacy when target DNA is present
False negative	Faulty or missing reagents	AIC-1^a^ or AIC-2^b^	Efficacy of reagents, PCR inhibitor activity (every reaction)
	PCR Inhibitors	AIC-1^a^ or AIC-2^b^	Efficacy of reagents, PCR inhibitor activity (every reaction)
	Faulty DNA extractions	HIC^c^	Successful DNA extraction, PCR inhibitor activity (every reaction)
	Faulty equipment	AIC-1^a^ or AIC-2^b^	Verify equipment efficiency
	Operator technique	AIC-1^a^ or AIC-2^b^ or HIC^c^	Verify operator performance

^a^For the AIC-1 control, no additional primer pair is required, the *R. toxicus* primers will also detect the AIC; ^b^For the AIC-2 control, one additional primer pair and probe (IC-PF2 and IC-PR2 with IC-P1 probe) is required to detect both AIC-2 and host genomic DNA (for host detection, additional probe IC-PLT will be required); ^c^For HIC, one additional primer set and probe (IC-PF2 and IC-PR2 with IC-PLT probe) is required.

High specificity and robustness of nucleic acid-based diagnostic methods, including qPCR and PCR-based assays, depend on appropriate target gene selection and primers/probes thermodynamics^9,16^. The highly conserved *R. toxicus* housekeeping genes showed only a few or no nucleotide differences among populations which made within-species population-level discrimination difficult to impossible^1^. Highly conserved housekeeping genes with high nucleotide similarity are common among bacterial species which limits their utility as diagnostic tools; it increases the possibilities of false positive results. Whole genome sequencing based diagnostic-target selection has become a superior approach for the identification of target genes that accurately discriminate among populations, strains or closely related species. Using whole genome sequence alignments, genomic regions unique to three populations (RT-I, RT-II and RT-III) of *R*. *toxicus* were identified upon which to develop population-specific assays. The three genomes showed more than 99.5% average nucleotide identity (ANI; Fig. 1). To detect all strains from all populations of *R. toxicus,* the *rpoD* gene was selected and provided a high degree of specificity as evidenced by the exclusivity panel. All the primers and probes were thermodynamically competent to achieve the highest sensitivity. Thermodynamically incompetent assays often result in low detection limits and may not be amenable to multiplex reactions^16,17^.

To establish the specificity of a detection assay, the validation procedure should include representative strains from all available populations (including genetic variants), closely related species^9,16,18^, and organisms found naturally in the same habitat as the target. The endpoint PCR and qPCR assays developed in this study showed no cross-reactivity with any member of the exclusivity panel comprised of four other species of the genus *Rathayibacter* and one species of a bacterium, *Dietzia cinnamea* SA03-14M, co-isolated from the same habitat as *R. toxicus* (Table 1). The PCR/qPCR assays successfully detected *R. toxicus* from all of the known *R. toxicus*-infected annual ryegrass samples, indicating no interference from host DNA or PCR inhibitors (Supplemental Table 1). Because many factors affect PCR efficiency and sensitivity, protocol detection limits and specificity were tested with four different master mix kits; two each for the endpoint and qPCR methods. Qiagen Multiplex Master Mix Kit and Qiagen Rotor-Gene Master Mix Kit exhibited consistent outcomes of detection limits for endpoint and qPCR assays, respectively. All four kits showed specific comparable results when single-target based sensitivity assays were performed. GoTaq Green Master Mix and Bio-Rad SsoFast Probes Supermix are recommended by the manufacturers to work best with single-target based assays; reaction costs for these kits are less than Qiagen multiplex kits (PCR and TaqMan qPCR), which makes them ideal for routine diagnostics. GoTaq Green Master Mix and Bio-Rad SsoFast Probes Supermix have also been used to develop sensitive multiplex PCR and multiplex TaqMan qPCR assays, respectively^11,19^.

Limit of detection is an important assay characteristic, especially for high consequence pathogens; low sensitivity can generate false negative outcomes, potentially resulting in a breach of biosecurity with high economic impacts. Based on quantitation using NanoDrop technology, the *R. toxicus* assays reported here detected to 1 fg of purified genomic DNA; approximately 0.41 genome copies. In direct comparisons with other technologies, NanoDrop was shown to be less sensitive at such low nucleic acid concentrations. The lower accuracy of NanoDrop quantitation may be the reason for the apparent detection to 1 fg which is equivalent to less than one genome copy. Consequently, the 1 fg detection limit reported in this study may be an over estimation of the actual analytic sensitivity of the described assays. Previously, Ouyang et al.^7^ developed a TaqMan qPCR-based bioforensic detection and discrimination method for *Xylella fastidiosa* subsp. *pauca*, a causal agent of citrus variegated chlorosis with a similar sensitivity of 1–10 fg. The *X. fastidiosa* subsp. *pauca* genome is approximately the same size (2.5 Mbp) as the *R. toxicus* genome (2.3 Mbp). Despite the variance in analytic sensitivity, the addition of internal controls to cross-check accuracy of DNA extraction and reagent efficiencies, make these protocols more reliable in term of accuracy and repeatability. Almost no differences were observed when sensitivity assays were performed with and without internal controls indicating the robustness of the assays. The assays reported here were tested for robustness by performing the assays by multiple operators in five labs in three countries with concordant results, no discrepancies were observed (Table 4).

The multigene endpoint and TaqMan qPCR-based detection assays for general and population-specific detection of *R. toxicus* using a variety of optional internal controls reported in this study are robust, accurate, reliable, and significantly sensitive; their use can speed phytosanitary diagnostics and subsequent decisions as well as routine pathogen detection. Accurate, reliable and early detection can facilitate to monitor infected samples and prevent pathogen dissemination through plant materials during interstate or international commerce. These developed assays have applications in routine diagnostics, biosecurity, surveillance, farm management and monitoring of resistance in plant breeding programs. The addition of internal controls enhanced the reliability of the developed assays; the approach can be more generally applied to develop assays for other plant pathogens.

## Materials and methods

### Sources of inclusivity and exclusivity panels, and DNA extraction

*R. toxicus* infected annual ryegrass samples were collected from Western Australia and South Australia during 2013–2014^1^. The inclusivity panel included 54 isolates of *R. toxicus* collected from different host plant species and different geographical locations in Australia over a 41-year period (1973 to 2014)^1^. The related species*, R. tritici, R. iranicus, R. rathayi, and R. agropyri* as well as *Dietzia cinnemea* (isolated from an *R. toxicus* bacterial gall) comprised the exclusivity panel. Isolates used in the inclusivity and exclusivity panels for both endpoint PCR and TaqMan real-time qPCR assay characterization were collected by the authors or were obtained from culture collections in Australia (Table 1). Each isolate was grown on 523 M medium and genomic DNA extracted using the DNeasy Blood & Tissue Kit (Cat. No. 69506; Qiagen, Valencia, CA) and/or Blood & Cell Culture DNA Midi Kit (Cat No. 13343; Qiagen) according to the manufacturer’s instructions (Arif et al., 2016). DNA from infected annual ryegrass was also extracted using the Qiagen DNeasy Blood & Tissue Kit. Genomic DNA concentration was measured using a NanoDrop 2000c spectrophotometer (Thermo Fisher Scientific Inc., Worcester, MA).

### Illumina MiSeq sequencing, assembly and annotation

Whole genome sequencing of representative isolates SA03-04 (population RT-I), SA08-07 (population RT-II), SAC7056 (population RT-II) and WAC3372 (population RT-III) was performed using Illumina MiSeq (Illumina, Inc., San Diego, CA) according to manufacturer’s instructions. The DNA paired-end libraries were prepared using the Nextera XT DNA Library Prep Kit following manufacturer’s instructions (Illumina, Inc.). One ng DNA from each isolate was used to prepare the library. Library fragment analysis was done using a 2100 Bioanalyser (Agilent Technologies, Santa Clara, CA). The assembled genomes were annotated using RAST (Rapid Annotation using Subsystem Technology)^20^.

### Target gene selection for primers and probes design

To select the target genes, the four representative genomes of the three genetic populations RT-I, RT-II and RT-III were aligned using Mauve^21^ and BRIG (BLAST Ring Image Generator)^22^. The complete genome of *R. toxicus* strain WAC3373 (Western Australia; NCBI GenBank accession number CP013292.1; size 2.346 MB) was used as a reference genome to align the genome assemblies (Fig. 1). Locally Collinear Blocks (LCBs) sequences generated by Mauve were annotated for target selection using Geneious version 9 (Biomatters, Inc. Newark, NJ). The Average Nucleotide Identity (ANI) of complete genomes was calculated using OrthoANI^23^. Endpoint PCR and qPCR assays for the detection of all *R. toxicus* isolates were designed using the highly conserved *rpoD* gene. *Rathayibacter toxicus* population-specific primers were designed based on genomic regions unique to each population (Fig. 1; Table 2). The primers and probes were designed using the online software Primer3^24^ following the parameters described by Arif and Ochoa-Corona (2013). Primer3 and mFold^25^ were used to predict the internal structure and the potential for self-dimer formation for each primer and probe. Details for all primers and probes are provided (Tables 2 and 3). Primers and probes were synthesized by IDT (Integrated DNA Technologies, Inc., Coralville, IA). Genome locations for primer and probe sites are provided (Supplement Fig. 3).

### Internal controls

Three internal controls were developed to enhance the reliability of the diagnostic assays and increase confidence in assay results: (1) A unique multi-target artificial internal control (AIC-1) was generated synthetically by adding the sense and anti-sense *R. toxicus*-specific primer sequences (RT-F1, RT-R1 and RT-R3) in an array into a plasmid cloning vector pIDTSmart (IDT). A universal probe (IC-P1) was designed to detect the AIC-1 amplicon; there was no 100% match for IC-P1 with any available nucleotide sequence in the NCBI GenBank nucleotide database). (2) An internal control (AIC-2) was designed that contained the sequences of a primer set that targeted the host genome (IC-PF2 and IC-PR2) and the universal probe (IC-P1) in a plasmid (cloning vector pUCIDTSmart) specifically designed to use as an internal control (3). A primer set and probe (HIC) were also designed and used to target the host genome (*L. rigidum*, annual ryegrass). Details of primers and probe used for internal controls are given in Tables 2 and 3 and Fig. 6.

### Single and multiplex endpoint PCR

Two different kits were used for endpoint PCR, GoTaq Green Master Mix (Cat. No. M7123; Promega, Madison, WI) and Qiagen Multiplex PCR Kit (Cat No. 206145; Qiagen). Four primer sets RT-F1/RT-R1 (product size 246 bp; to detect all *R. toxicus*), RT-AF1/RT-AR1 (product size 411 bp; to detect only *R. toxicus* population RT-I), RT-BF1/RT-BR1 (product size 532 bp; to detect only *R. toxicus* population RT-II), and RT-CF1/RT-CR1 (product size 677 bp; to detect only *R. toxicus* population RT-III) were used in multiplex PCR. Different internal control primer sets were also used to detect host DNA and/or plasmid DNA (Table 2). Plasmid DNA (AIC-1; artificial internal control) containing the target sequences for primer set RT-F1/RT-R1 was synthesized and used as an internal control without adding any additional primers in the reaction. However, the primer set RT-F1/RT-R1concentration was increased in the reaction by 1.5-fold. Different PCR conditions were used for the different kits (GoTaq Green Master Mix and Qiagen Multiplex PCR Kit). The assays were performed with and without internal controls to compare the results to confirm specificity and reliability (Supplemental Table 3). Different PCR components and conditions used for different assays are presented Supplemental Table 4. The amplified products were electrophoresed on 1.5% agarose gel using TAE buffer. A 50-bp ladder was used to determine the amplicons size. Bio-Rad DNA Engine (Bio-Rad, Hercules, CA), PTC-200 Gradient Thermal Cycler (MJ Research Inc., Watertown, MA) and Eppendorf™ Mastercycler™ pro PCR System (Hamburg, Germany) were used to perform the assays.

### Single and multiplex TaqMan real-time qPCR

TaqMan real-time qPCR single and multiplex assays were also performed using two kits—SsoFast Probes Supermix (Cat. No. 172-5231; Bio-Rad) and Rotor-Gene Multiplex PCR Master Mix (Cat No. 204774; Qiagen). Primer set RT-F1/RT-R3 and probe RT-P1 (product size 115 bp) were used to detect all populations of *R. toxicus.* TaqMan real-time qPCR assays were also performed with and without internal controls. The primers and probe sequences for internal controls are mentioned in Tables 2 and 3. Different qPCR conditions were used for two different kits (SsoFast Probes Supermix and Rotor-Gene Multiplex PCR Master Mix). Each reaction was performed in three replicates and a CFX96 qPCR machine (Bio-Rad, Hercules, CA) was used to run the assays. The data was analyzed using Bio-Rad CFX Manager 3.1. TaqMan real-time qPCR conditions and components for different assays are presented in Supplemental Table 4. Cycle threshold was calibrated automatically.

### Sensitivity assays

The sensitivity and efficiency^26^ of each primer set with and without internal controls were assessed in endpoint PCR and TaqMan real-time qPCR. A tenfold serial dilution of *R. toxicus* genomic DNA was used to perform the sensitivity and spiked sensitivity (added 10 ng of *L. rigidum* genomic DNA in each reaction) assays. The sensitivity assays were performed in different combinations of *R. toxicus*-specific primers/probes with different primers/probe sets targeting different internal controls. Details are mentioned in Supplemental Table 2 and Fig. 6. In both endpoint and TaqMan real-time qPCR assays, 10 pg of plasmid internal control was used. All four kits (2 each for endpoint PCR and TaqMan real-time qPCR) were used for the sensitivity assays. Each qPCR reaction was performed in three replicates.

### Blind/ring tests

Genomic DNA of seven *R. toxicus* strains and NTC (water) were used to perform the blind tests at two national (USA) and three international labs (Australia and New Zealand). Blind tests for multiplex endpoint PCR and TaqMan real-time qPCR in four and three labs, respectively were performed by multiple operators at different time periods (Table 4). All tests were performed with an artificial internal control (AIC-1). All qPCR reactions were performed in three replicates. In two labs (Elizabeth Macarthur Agricultural Institute, Menangle, NSW, Australia and La Trobe University, Bundoora, VIC, Australia), TaqMan real-time qPCR assays were performed in Corbett Rotor-Gene 6000 thermocyclers (Corbett Research, Sydney, Australia) while the other two labs (Kansas State University Manhattan, KS and Plant & Food Research, Lincoln, New Zealand) used Bio-Rad CFX96 engines to perform the qPCR assays. Qiagen Multiplex PCR and Rotor-Gene Multiplex PCR Master Mix kits were used for endpoint and qPCR multiplex assays, respectively.

### Detection from infected samples of annual rye grass

Seed of annual rye grass containing infected florets were placed into 15 ml tubes with marbles, filled with sterile water to the 20 ml mark, and soaked for 40 min; tubes were shaken vigorously for 1 min at 20 min intervals. The supernatant (2 ml) was transferred to a 2 ml eppendorf tube and centrifuged at 8000 rpm for 5 min; genomic DNA was extracted from the pellets using Qiagen DNeasy Blood & Tissue Kit according to the manufacturer’s instructions. Endpoint multiplex and TaqMan qPCR assays with internal control AIC-1 were performed; samples information is given in Supplemental Table 1.

### Detection and discrimination of *R. toxicus* and their populations directly from live cultures

Eight cultures of *R. toxicus* and one culture of each species of *Rathayibacter* (*R. tritici. R. agropyri, R. iranicus* and *R. rathayi*) were taken and boiled separately in 100 µl of sterile water at 100 °C for 5 min followed by centrifugation for 2 min at 6000 rpm. Supernatant was transferred to clean tubes and 1 µl was used for each endpoint PCR and TaqMan real-time qPCR. PCR and qPCR conditions and components were used as mentioned in Supplemental Table 4. Each reaction was run with internal control AIC-1. This experiment was performed at La Trobe University, Bundoora, VIC, Australia. Details are given in Supplemental Table 5.

## Supplementary Information

Supplementary Information

## Data Availability

All sequencing data is available in NCBI GenBank database.
