# Supplementary material for: Multiple internal controls enhance reliability for PCR and real time PCR detection of Rathayibacter toxicus
Source: Sci Rep. 2021 Apr 16;11:8365. doi: 10.1038/s41598-021-87815-6 (PMC8052354; doi:10.1038/s41598-021-87815-6)
Supplement: Supplementary file 1 — Supplementary Information [file 41598_2021_87815_MOESM1_ESM.docx]

**Supplementary Information**

**Multiple internal controls enhance reliability for PCR and real time PCR detection of *Rathayibacter toxicus***

Mohammad Arif^1,2,4^, Grethel Y. Busot^2,4^, Rachel Mann^3,4^, Brendan Rodoni^3,4^ and

James P. Stack^2,4*^

^1^Department of Plant and Environmental Protection Sciences, University of Hawaii at Manoa, Honolulu, Hawaii, USA; ^2^Department of Plant Pathology, Kansas State University, Manhattan, Kansas, USA; ^3^Department of Economic Development, Jobs, Transport and Resources, Biosciences Research Division, Bundoora, Victoria, Australia. ^4^Plant Biosecurity Cooperative Research Centre, Canberra, Australia

*Communicating Author: email - jstack@ksu.edu

**Running title**

Detection and discrimination of *Rathayibacter toxicus* populations

*
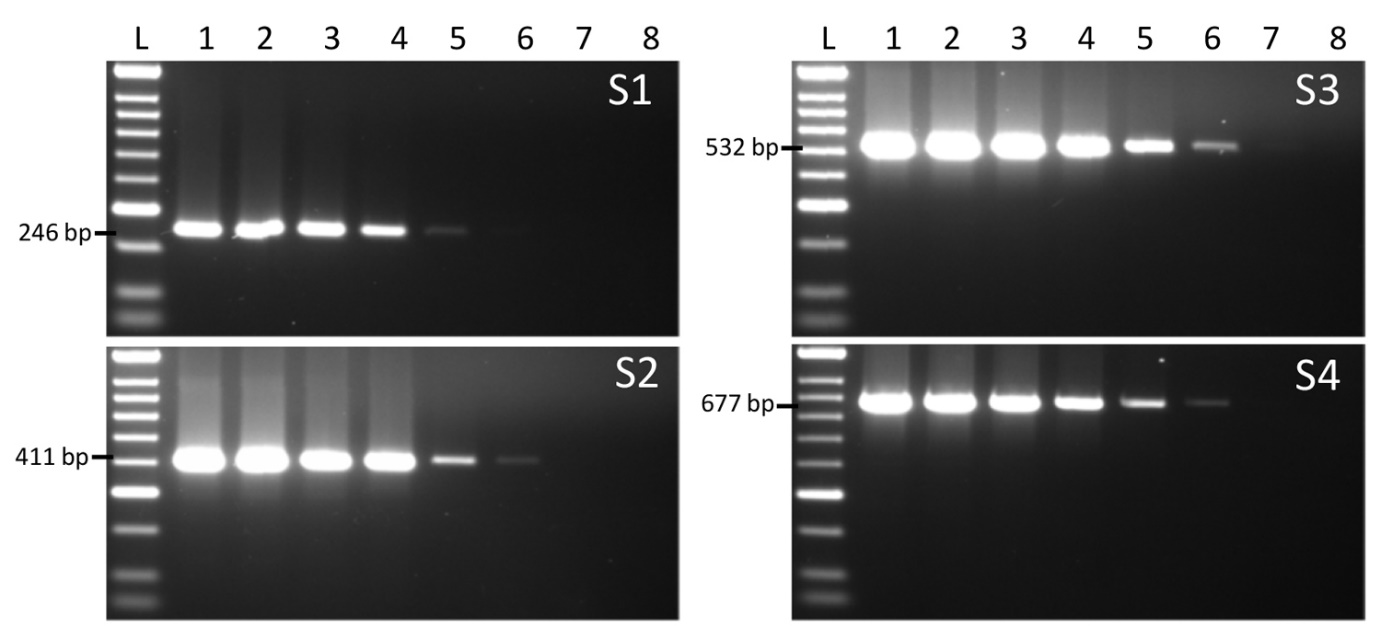
*

**Supplemental Figure 1.** **Singleplex PCR with GoTaq Green Master Mix to detect *Rathayibacter toxicus*.** S1. to detect all *R. toxicus*; S2. to detect *R. toxicus* population RT-I; S3. to detect *R. toxicus* population RT-II; S4. to detect *R. toxicus* population RT-III. Lane 1 –7 are genomic DNA dilutions from 1 ng to 1 fg; lane 8 is non-template control (NTC; water)


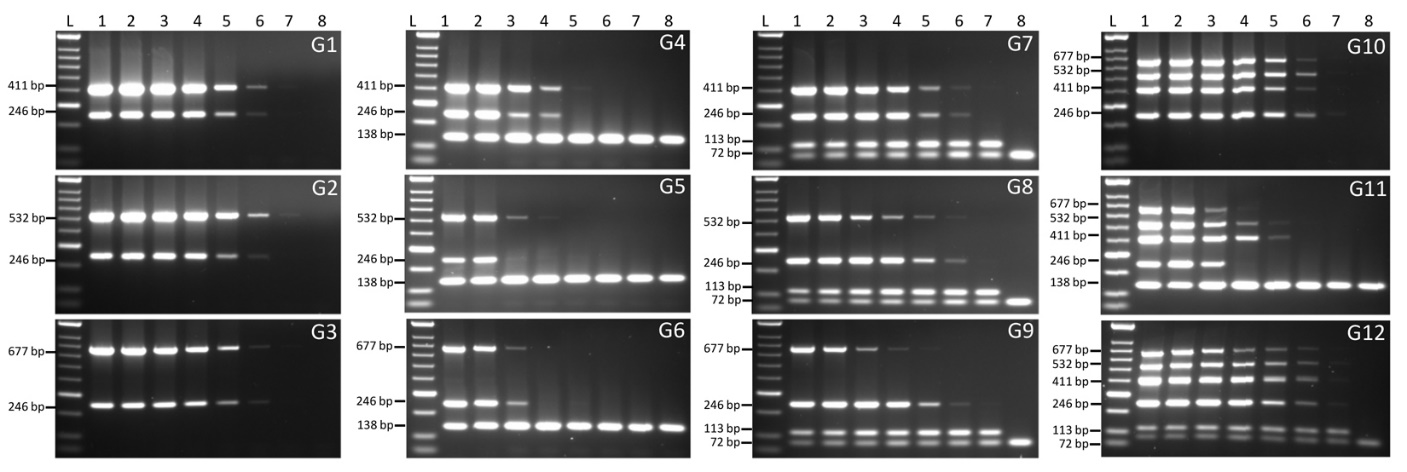


**Supplemental Figure 2.** **Comparative sensitivity assays to predict the detection limit of *Rathayibacter toxicus* using multiplex endpoint PCR with and without internal controls.** GoTaq Green Master Mix was used for these sensitivity assays. G1. DNA from population RT-I only + No AIC; G2. DNA from population RT-II only + No AIC; G3. DNA from population RT-III only + No AIC; G4. DNA from population RT-I only + AIC-1; G5. DNA from population RT-II only + AIC-1; G6. DNA from population RT-III only + AIC-1; G7. DNA from population RT-I only + AIC-2 & HIC; G8. DNA from population RT-II only + AIC-2 & HIC; G9. DNA from population RT-III only + AIC-2 & HIC; G10. DNA from all three populations + No AIC; G11. DNA from all three populations + AIC-1; G12. DNA from all three populations + AIC-2 & HIC. Lane 1 –7 are genomic DNA dilutions from 1 ng to 1 fg; lane 8 is non-template control (NTC; water)


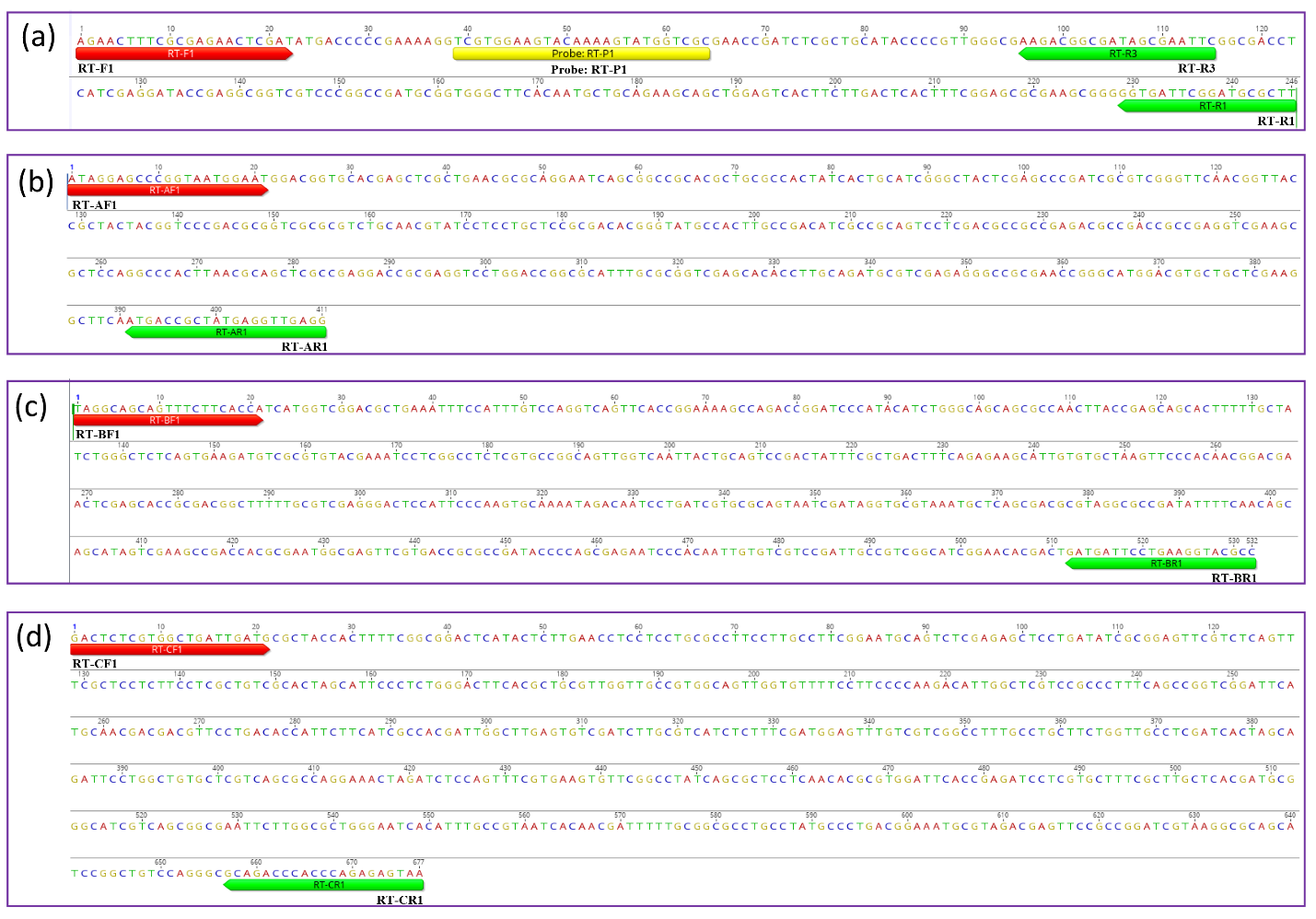


**Supplemental Figure 3.** ***Rathayibacter toxicus* target genome regions and primers/probes locations.** (a) Target regions *rpoD* for primer and probe to detect all *R. toxicus*; (b) target regions Intergenic and Transcriptional regulator, MerR family for primer set to detect *R. toxicus* population RT-I; (c) target region Intergenic & hypothetical gene for primer set to detect *R. toxicus* population RT-II; (d) target region NA(+)/H(+) antiporter gene for primer set to detect *R. toxicus* population RT-III.

**Supplemental Table 1.** Detection and discrimination of *Rathayibacter toxicus* from infected annual rye grass samples using multiplex endpoint PCR and TaqMan real-time qPCR-based methods.

| Sample number | Location | Year of collection | Endpoint PCR results | | | qPCR results | |
| --- | --- | --- | --- | --- | --- | --- | --- |
|  |  |  | *R. toxicus* | Population identified | AIC-1 | *R. toxucs* | AIC-1 |
| SA03 | South Australia | 2014 | + | RT-I | + | + | + |
| SA08 | South Australia | 2014 | + | RT-I | + | + | + |
| SA19 | South Australia | 2014 | + | R-T-I & RT-II | + | + | + |
| SA70 | South Australia | 2014 | - | - | + | - | + |
| WA06 | Western Australia | 2014 | - | - | + | - | + |
| WA08 | Western Australia | 2014 | - | - | + | - | + |
| WA41 | Western Australia | 2014 | - | - | + | - | + |
| WA61 | Western Australia | 2014 | - | - | + | - | + |
| WA68 | Western Australia | 2014 | - | - | + | - | + |
| WA69 | Western Australia | 2014 | - | - | + | - | + |
| Positive control | - | - | + | RT-II | + | + | + |
| Water | - | - | - | - | + | - | + |

AIC-1 = artificial internal control

**Supplemental Table 2.** Detail of different sensitivity assays performed with multiplex endpoint PCR.

| Code | Sensitivity assay parameters | PCR/qPCR kit used |
| --- | --- | --- |
| E1 | DNA from population RT-I only + No AIC | Qiagen kit |
| E2 | DNA from population RT-II only + No AIC | Qiagen kit |
| E3 | DNA from population RT-III only + No AIC | Qiagen kit |
| E4 | DNA from population RT-I only + AIC-1 | Qiagen kit |
| E5 | DNA from population RT-II only + AIC-1 | Qiagen kit |
| E6 | DNA from population RT-III only + AIC-1 | Qiagen kit |
| E7 | DNA from population RT-I only + AIC-2 & HIC | Qiagen kit |
| E8 | DNA from population RT-II only + AIC-2 & HIC | Qiagen kit |
| E9 | DNA from population RT-III only + AIC-2 & HIC | Qiagen kit |
| E10 | DNA from all three populations + No AIC | Qiagen kit |
| E11 | DNA from all three populations + AIC-1 | Qiagen kit |
| E12 | DNA from all three populations + AIC-2 & HIC | Qiagen kit |
| S1 | *R. toxicus* DNA + only primer set RT-F1/RT-R1 | GoTaq kit |
| S2 | *R. toxicus* RT-I DNA + only primer set RT-AF1/RT-AR1 | GoTaq kit |
| S3 | *R. toxicus* RT-II DNA + only primer set RT-BF1/RT-BR1 | GoTaq kit |
| S4 | *R. toxicus* RT-III DNA + only primer set RT-CF1/RT-CR1 | GoTaq kit |
| G1 | DNA from population RT-I only + No AIC | GoTaq kit |
| G2 | DNA from population RT-II only + No AIC | GoTaq kit |
| G3 | DNA from population RT-III only + No AIC | GoTaq kit |
| G4 | DNA from population RT-I only + AIC-1 | GoTaq kit |
| G5 | DNA from population RT-II only + AIC-1 | GoTaq kit |
| G6 | DNA from population RT-III only + AIC-1 | GoTaq kit |
| G7 | DNA from population RT-I only + AIC-2 & HIC | GoTaq kit |
| G8 | DNA from population RT-II only + AIC-2 & HIC | GoTaq kit |
| G9 | DNA from population RT-III only + AIC-2 & HIC | GoTaq kit |
| G10 | DNA from all three populations + No AIC | GoTaq kit |
| G11 | DNA from all three populations + AIC-1 | GoTaq kit |
| G12 | DNA from all three populations + AIC-2 & HIC | GoTaq kit |

AIC – artificial internal control (target inserted into plasmid); HIS – host internal control (*Lolium rigidum* ITS region was targeted); RT-I, RT-II & RT-III are *R. toxicus* populations

**Supplemental Table 3.** Details of inclusivity and exclusivity panels used to validate the endpoint PCRs with and without internal control (AIC-1) using GoTaq master mix

| Genus/Species | Isolate Code | Multiplex with AIC-1 | | Multiplex without AIC-1 | | All RT primer set with AIC | Primer set only for RT-I | Primer set only for RT-II | Primer set only for RT-III |
| --- | --- | --- | --- | --- | --- | --- | --- | --- | --- |
|  |  | Multiplex PCR | Population identified | Multiplex PCR | Population identified |  |  |  |  |
| *Rathayibacter toxicus* | SA03-02 | + | RT-I | + | RT-I | + | + | - | - |
| *R. toxicus* | SA03-03 | + | RT-I | + | RT-I | + | + | - | - |
| *R. toxicus* | SA03-04 | + | RT-I | + | RT-I | + | + | - | - |
| *R. toxicus* | SA03-08 | + | RT-I | + | RT-I | + | + | - | - |
| *R. toxicus* | SA03-14 | + | RT-I | + | RT-I | + | + | - | - |
| *R. toxicus* | SA03-15 | + | RT-I | + | RT-I | + | + | - | - |
| *R. toxicus* | SA03-16 | + | RT-I | + | RT-I | + | + | - | - |
| *R. toxicus* | SA03-17 | + | RT-I | + | RT-I | + | + | - | - |
| *R. toxicus* | SA03-18 | + | RT-I | + | RT-I | + | + | - | - |
| *R. toxicus* | SA03-19 | + | RT-I | + | RT-I | + | + | - | - |
| *R. toxicus* | SA03-20 | + | RT-I | + | RT-I | + | + | - | - |
| *R. toxicus* | SA03-21 | + | RT-I | + | RT-I | + | + | - | - |
| *R. toxicus* | SA03-22 | + | RT-I | + | RT-I | + | + | - | - |
| *R. toxicus* | SA03-23 | + | RT-I | + | RT-I | + | + | - | - |
| *R. toxicus* | SA03-24 | + | RT-I | + | RT-I | + | + | - | - |
| *R. toxicus* | SA03-25 | + | RT-I | + | RT-I | + | + | - | - |
| *R. toxicus* | SA03-26 | + | RT-I | + | RT-I | + | + | - | - |
| *R. toxicus* | SA03-27 | + | RT-I | + | RT-I | + | + | - | - |
| *R. toxicus* | SA03-28 | + | RT-I | + | RT-I | + | + | - | - |
| *R. toxicus* | SA08-03 | + | RT-I | + | RT-I | + | + | - | - |
| *R. toxicus* | SA08-07 | + | RT-II | + | RT-II | + | - | + | - |
| *R. toxicus* | SA08-08 | + | RT-I | + | RT-I | + | + | - | - |
| *R. toxicus* | SA08-09 | + | RT-I | + | RT-I | + | + | - | - |
| *R. toxicus* | SA08-11 | + | RT-I | + | RT-I | + | + | - | - |
| *R. toxicus* | SA08-13 | + | RT-I | + | RT-I | + | + | - | - |
| *R. toxicus* | SA08-16 | + | RT-I | + | RT-I | + | + | - | - |
| *R. toxicus* | SA19-02 | + | RT-I | + | RT-I | + | + | - | - |
| *R. toxicus* | SA19-03 | + | RT-II | + | RT-II | + | - | + | - |
| *R. toxicus* | SA19-04 | + | RT-I | + | RT-I | + | + | - | - |
| *R. toxicus* | SA19-05 | + | RT-II | + | RT-II | + | - | + | - |
| *R. toxicus* | SA19-06 | + | RT-I | + | RT-I | + | + | - | - |
| *R. toxicus* | SA19-07 | + | RT-I | + | RT-I | + | + | - | - |
| *R. toxicus* | SA19-08 | + | RT-II | + | RT-II | + | - | + | - |
| *R. toxicus* | SA19-09 | + | RT-I | + | RT-I | + | + | - | - |
| *R. toxicus* | SA19-10 | + | RT-II | + | RT-II | + | - | + | - |
| *R. toxicus* | SA19-11 | + | RT-I | + | RT-I | + | + | - | - |
| *R. toxicus* | SA19-12 | + | RT-I | + | RT-I | + | + | - | - |
| *R. toxicus* | SA19-13 | + | RT-I | + | RT-I | + | + | - | - |
| *R. toxicus* | SA19-14 | + | RT-II | + | RT-II | + | - | + | - |
| *R. toxicus* | SAC3368 | + | RT-II | + | RT-II | + | - | + | - |
| *R. toxicus* | SAC3387 | + | RT-II | + | RT-II | + | - | + | - |
| *R. toxicus* | SAC7056 | + | RT-II | + | RT-II | + | - | + | - |
| *R. toxicus* | WAC3371 | + | RT-III | + | RT-III | + | - | - | + |
| *R. toxicus* | WAC3372 | + | RT-III | + | RT-III | + | - | - | + |
| *R. toxicus* | WAC3373 | + | RT-III | + | RT-III | + | - | - | + |
| *R. toxicus* | WAC3396 | + | RT-III | + | RT-III | + | - | - | + |
| *R. toxicus** | FH100 | + | RT-II | + | RT-II | + | - | + | - |
| *R. toxicus** | FH83 | + | RT-II | + | RT-II | + | - | + | - |
| *R. toxicus** | FH85 | + | RT-II | + | RT-II | + | - | + | - |
| *R. toxicus** | FH147 | + | RT-II | + | RT-II | + | - | + | - |
| *R. toxicus** | FH141 | + | II | + | RT-II | + | - | + | - |
| *R. toxicus** | FH81 | + | III | + | RT-III | + | - | - | + |
| *R. toxicus** | FH138 | + | RT-III | + | RT-III | + | - | - | + |
| *R. toxicus** | FH87 | + | RT-III | + | RT-III | + | - | - | + |
| *R. tritici* | WAC7055 | - | - | - | - | - | - | - | - |
| *R. tritici* | WAC9601 | - | - | - | - | - | - | - | - |
| *R. tritici* | WAC9602 | - | - | - | - | - | - | - | - |
| *R. agropyri* | WAC9620 | - | - | - | - | - | - | - | - |
| *R. agropyri* | WAC9594 | - | - | - | - | - | - | - | - |
| *R. iranicus* | ICMP 12831 | - | - | - | - | - | - | - | - |
| *R. iranicus* | ICMP 13126 | - | - | - | - | - | - | - | - |
| *R. iranicus* | ICMP 13127 | - | - | - | - | - | - | - | - |
| *R. iranicus* | ICMP 3496 | - | - | - | - | - | - | - | - |
| *R. rathayi* | ICMP 2579 | - | - | - | - | - | - | - | - |
| *R. rathayi* | ICMP 2574 | - | - | - | - | - | - | - | - |
| *R. rathayi* | WAC3369 | - | - | - | - | - | - | - | - |
| *D. cinnamea* | SA03-14M | - | - | - | - | - | - | - | - |

*Only DNA was available for this study; AIC-artificial internal control.

**Supplemental Table 4.** Endpoint PCR and qPCR conditions and components used for detection and discrimination of *Rathayibacter toxicus* from pure culture and infected plant materials.

| Protocol Number | PCR components  (per reaction) | PCR conditions | Targets |
| --- | --- | --- | --- |
| PCR-1 | GoTaq Green Master Mix = 25 µL  *Primer F = 2.0 µL  *Primer R = 2.0 µL  Water = 20.0 µL  DNA Template = 1.0 µL  **Total Volume = 50.0 µL**  Note: PCR with half amount of PCR components works equally good | 1. Initial denaturation 95 ⁰C for 5 min  2. Denaturation 95 ⁰C for 20 s  3. Annealing 59⁰C for 45 s  4. Extension 72⁰C for 45 s  5. Final extension 72⁰C for 3 min  (total 35 cycles) | *R. toxicus* in general.  RT-I, RT-II and RT-III populations of *R. toxicus* |
| PCR-2 | GoTaq Green Master Mix = 25 µL  **Primer Mix 2 = 2.0 µL  Water = 20.0 µL  DNA Template = 1.0 µL  **Total Volume = 50.0 µL** | 1. Initial denaturation 95 ⁰C for 5 min  2. Denaturation 95 ⁰C for 20 s  3. Annealing 59⁰C for 45 s  4. Extension 72⁰C for 45 s  5. Final extension 72⁰C for 3 min  (total 35 cycles) | Multiplex for *R. toxicus* in general and all the populations |
| PCR-3 | GoTaq Green Master Mix = 25 µL  ***Primer Mix 3 = 2.0 µL  Water = 20.0 µL  DNA Template = 1.0 µL  ^#^(Internal control -10 pg)  **Total Volume = 50.0 µL** | 1. Initial denaturation 95 ⁰C for 5 min  2. Denaturation 95 ⁰C for 20 s  3. Annealing 59⁰C for 45 s  4. Extension 72⁰C for 45 s  5. Final extension 72⁰C for 3 min  (total 35 cycles) | Multiplex for *R. toxicus* in general and all the populations plus internal control(s) |
| PCR-4 | Qiagen Multiplex Kit = 25 µL  **Primer Mix 2 = 2.0 µL  Water = 20.0 µL  DNA Template = 1.0 µL  **Total Volume = 50.0 µL** | 1. Initial denaturation 95⁰C for 15 min  2. Denaturation 95⁰C for 30 s  3. Annealing 72⁰C for 45 s  4. Extension 59⁰C for 45 s  5. Final extension 72⁰C for 5 min  (total 35 cycles) | Multiplex for *R. toxicus* in general and all *R. toxicus* populations |
| PCR-5 | Qiagen Multiplex Kit = 25 µL  ***Primer Mix 3 = 2.0 µL  Water = 20.0 µL  DNA Template = 1.0 µL  ^#^(Internal control -10 pg)  **Total Volume = 50.0 µL** | 1. Initial denaturation 95⁰C for 15 min  2. Denaturation 95⁰C for 30 s  3. Annealing 72⁰C for 45 s  4. Extension 59⁰C for 45 s  5. Final extension 72⁰C for 5 min  (total 35 cycles) | Multiplex for *R. toxicus* in general and all the populations plus internal control(s) |
| PCR-6 | Qiagen Multiplex Kit = 25 µL  ***Primer Mix 4 = 2.0 µL  Water = 20.0 µL  DNA Template = 1.0 µL  Host DNA = 1.0 µL  ^#^(AIC-2 = 10 pg)  **Total Volume = 50.0 µL** | 1. Initial denaturation 95⁰C for 15 min  2. Denaturation 95⁰C for 30 s  3. Annealing 72⁰C for 45 s  4. Extension 59⁰C for 45 s  5. Final extension 72⁰C for 5 min  (total 35 cycles) | Multiplex for *R. toxicus* in general and all the populations plus internal control(s) |
|  |  |  |  |
| **TaqMan Real time qPCR Protocols** | | | |
| qPCR-6 | Sso SuperFast Master Mix = 12.5 µL  Primer RT-F1 = 1.0 µL  Primer RT-R3 = 1.0 µL  Probe RT-P1 = 1.0 µL  Water = 8.5 µL  DNA Template = 1.0 µL  **Total Volume = 25.0 µL** | 1. Initial denaturation 95 ⁰C for 3 min  2. Denaturation 95 ⁰C for 15 s  3. Annealing/extension 59⁰C for 45 s (use only 30 s for one target)  (total 40 cycles) | *R. toxicus* |
| qPCR-7 | Qiagen Master Mix = 12.5 µL  Primer RT-F1 = 1.0 µL  Primer RT-R1 = 1.0 µL  Primer IC-PF2 = 1.0 µL  Primer IC-PR2= 1.0 µL  Probe RT-P1 = 1.0 µL  Probe IC-PLT = 1.0 µL  Water = 5.5 µL  DNA Template = 1.0 µL  **Total volume = 25.0** **µL** | 1. Initial denaturation 95 °C for 5 min  2. Denaturation 95 °C for 30 s  3. Annealing/extension 60°C for 45 s  (total 40 cycles) | *R. toxicus* and host genome |
| qPCR-8 | Qiagen Master Mix = 12.5 µL  Primer RT-F1 = 1.0 µL  Primer RT-R1 = 1.0 µL  Primer IC-PF2 = 1.0 µL  Primer IC-PR2= 1.0 µL  Probe RT-P1 = 1.0 µL  Probe IC-PLT = 1.0 µL  Water = 5.5 µL  DNA Template = 1.0 µL  **Total volume = 25.0** **µL** | 1. Initial denaturation 95 °C for 5 min  2. Denaturation 95 °C for 30 s  3. Annealing/extension 60°C for 45 s  (total 40 cycles) | *R. toxicus* and host genome |

*Forward and reverse primers are based on target (*R. toxicus* in general or population specific). ^#^ten pg plasmid artificial internal control (AIC-1 or AIC-2) was added.

Primer Mix 2 = 10 µL of each primer (RT-F1, RT-R1, RT-AF1, RT-AR1, RT-BF1, RT-BR1, RT-CF1, RT-CR1,) from 100 µM stock plus 120 µL water. 0.2 µM each primer concentration in 50 µL reaction.

Primer Mix 3 = 15 µL of each primer RT-F1, RT-R1 and 10 µL of each primer (RT-F1, RT-R1, RT-AF1, RT-AR1, RT-BF1, RT-BR1, RT-CF1, RT-CR1,) from 100 µM stock plus 110 µL water. 0.3 µM of primer RT-F1 and RT-R1, and 0.2 µM concentration of rest primer (each) in 50 µL reaction.

Primer Mix 4 = 10 µL of each primer (RT-F1, RT-R1, RT-AF1, RT-AR1, RT-BF1, RT-BR1, RT-CF1, RT-CR1, IC-PF2, IC-PR2) from 100 µM stock plus 100 µL water. 0.2 µM each primer concentration in 50 µL reaction.

**Supplemental Table 5.** Detection and discrimination of *Rathayibacter toxicus* using colony PCR (boiled followed by short spin) using multiplex endpoint PCR and TaqMan real-time qPCR-based methods.

| Isolate number | Name | Year of collection | Region/Country | Detection and discrimination results | | qPCR results | |
| --- | --- | --- | --- | --- | --- | --- | --- |
|  |  |  |  | Population identified as | AIC-1 | *R. toxicus* | AIC-1 |
| WAC3387 | *Rathayibacter toxicus* | 1981 | South Australia | RT-II | + | + | + |
| SA08-07 | *R. toxicus* | 2014 | South Australia | RT-II | + | + | + |
| WAC3372 | *R. toxicus* | 1978 | Western Australia | RT-III | + | + | + |
| SA08-13 | *R. toxicus* | 2014 | South Australia | RT-I | + | + | + |
| CS28/FH138 | *R. toxicus* | 1978 | Western Australia | RT-III | + | + | + |
| SA03-22 | *R. toxicus* | 2014 | South Australia | RT-I | + | + | + |
| SA03-25 | *R. toxicus* | 2014 | South Australia | RT-I | + | + | + |
| WAC9601 | *Rathayibacter tritici* | - | Western Australia | - | + | - | + |
| WAC9620 | *Rathayibacter agropyri* | - | Western Australia | - | + | - | + |
| ICMP13126 | *Rathayibacter iranicus* | 1994 | Iran | - | + | - | + |
| ICMP2579 | *Rathayibacter rathayi* | - | United Kingdom | - | + | - | + |
| NTC | Water | - | - | - | + | - | + |

AIC-1 = artificial internal control
